# The Effect of Vascular Segmentation Methods on Stereotactic Trajectory Planning for Drug-Resistant Focal Epilepsy: A Retrospective Cohort Study

**DOI:** 10.1016/j.wnsx.2019.100057

**Published:** 2019-10

**Authors:** Vejay N. Vakharia, Rachel Sparks, Sjoerd B. Vos, Andrew W. McEvoy, Anna Miserocchi, Sebastien Ourselin, John S. Duncan

**Affiliations:** 1 Department of Clinical and Experimental Epilepsy, UCL Institute of Neurology, University College London, London, United Kingdom; 2 Epilepsy Society MRI Unit, Chalfont St. Peter, United Kingdom; 3 Wellcome/EPSRC Centre Interventional and Surgical Sciences, University College London, London, United Kingdom; 4 Translational Imaging Group, Centre for Medical Image Computing, University College London, London, United Kingdom; 5 National Hospital for Neurology and Neurosurgery, Queen Square, London, United Kingdom; 6 School of Biomedical Engineering and Imaging Sciences, St Thomas’ Hospital, King’s College London, London, United Kingdom

**Keywords:** Computer-assisted planning, Epilepsy, EpiNav, Stereoelectroencephalography, Vascular segmentation

## Abstract

**Background:**

Stereotactic neurosurgical procedures carry a risk of intracranial hemorrhage, which may result in significant morbidity and mortality. Vascular imaging is crucial for planning stereotactic procedures to prevent conflicts with intracranial vasculature. There is a wide range of vascular imaging methods used for stereoelectroencephalography (SEEG) trajectory planning. Computer-assisted planning (CAP) improves planning time and trajectory metrics. We aimed to quantify the effect of different vascular imaging protocols on CAP trajectories for SEEG.

**Methods:**

Ten patients who had undergone SEEG (95 electrodes) following preoperative acquisition of gadolinium-enhanced magnetic resonance imaging (MR + Gad), magnetic resonance angiography and magnetic resonance angiography (MRV + MRA), and digital subtraction catheter angiography (DSA) were identified from a prospectively maintained database. SEEG implantations were planned using CAP using DSA segmentations as the gold standard. Strategies were then recreated using MRV + MRA and MR + Gad to define the “apparent” and “true” risk scores associated with each modality. Vessels of varying diameter were then iteratively removed from the DSA segmentation to identify the size at which all 3 vascular modalities returned the same safety metrics.

**Results:**

CAP performed using DSA vessel segmentations resulted in significantly lower “true” risk scores and greater minimum distances from vasculature compared with the “true” risk associated with MR + Gad and MRV + MRA. MRV + MRA and MR + Gad returned similar risk scores to DSA when vessels <2 mm and <4 mm were not considered, respectively.

**Conclusions:**

Significant variability in vascular imaging and trajectory planning practices exist for SEEG. CAP performed with MR + Gad or MRV + MRA alone returns “falsely” lower risk scores compared with DSA. It is unclear whether DSA is oversensitive and thus restricting potential trajectories.

## Introduction

Stereoelectroencephalography (SEEG) is a procedure in which multiple electrodes (typically 8–14) are stereotactically inserted into predefined brain regions as part of the presurgical evaluation of patients with drug-resistant focal epilepsy.^1^ The greatest risk of this and other stereotactic intracerebral procedures, such as deep brain stimulation, tumor biopsy, and laser ablation, is hemorrhage. A recent meta-analysis of SEEG procedures found that on average 10 electrodes were implanted in each patient and the overall rate of morbidity was 1 in 287 electrodes, equating to 1 in 29 patients implanted.^2^ All feasible measures to reduce the risk of hemorrhage should be considered and implemented if possible. To this end, surgeons plan electrode trajectories to pass through avascular corridors to prevent collision with intracranial vessels.

Given the low rate of hemorrhage, prohibitively large sample sizes would be needed to undertake a comparative study between vascular imaging techniques. Surrogate measures of hemorrhage risk have been used in previous studies as a pragmatic compromise.^3^, ^4^, ^5^ To this end, “risk scores” (RS) are calculated preoperatively for each electrode as a mathematical representation of the size of the avascular corridor, calculated through cumulative distance measurements from intracranial vasculature along the entire trajectory. Electrode planning can be undertaken manually or using computer-assisted planning (CAP).^6^, ^7^ CAP recently has been shown to reduce RS compared with manually planned electrodes while also minimizing the intracerebral length, white matter sampling, drilling angle to the skull, and overall planning time.^4^ Ratings of electrode feasibility by blinded external experts have shown no significant difference between manual and CAP trajectories when digital subtraction catheter angiography (DSA) was implemented during planning.^4^

A major disadvantage of calculated RS is that it is dependent on the method of vascular imaging and the vascular segmentation protocol used. Gadolinium-enhanced T1 magnetic resonance imaging (MR + Gad) images are critical for electrode planning. Due to the low contrast-to-noise ratio, however, it is not possible to segment the majority of the visible vessels, even with the appropriate use of filters,^8^ for use in the calculation of the RS. Further methods of vascular imaging include magnetic resonance venography (MRV), magnetic resonance angiography (MRA),^4^, ^9^ and DSA.^10^ Our objective is to quantify the effect that different vascular segmentation methods have on RS, and therefore CAP planning, in patients who have undergone MR + Gad, MRV + MRA, and DSA, with DSA as the gold standard.

## Methods

### Participants

Ten consecutive patients (95 electrodes) who had MR + Gad, MRV, MRA, and DSA images between 2016 and 2018 were selected from a prospectively maintained database of SEEG implantations. All patients consented for the data to be used in scientific research and publication. National Research Ethics Service Committee London approval reference: 12/LO/0377.

### Image Acquisition

#### Magnetic Resonance Imaging (MRI)

All MRI data were acquired on an MR750 3T (GE Healthcare, Madison, Wisconsin, USA), with a maximum gradient strength of 50 mT/m and maximum slew rate of 200 T/m/s, with a 32-channel head coil. For further information see the Supplementary Material.

#### Digital Subtraction Catheter Angiography

All DSA images were undertaken through internal carotid and vertebral artery injections in an interventional radiology suite using a bi-plane C-arm for bi-planar fluoroscopy and rotational 3-dimensional angiography. For further information, see the Supplementary Material.

### Model Generation

A whole brain parcellation was generated from a volumetric T1-weighted magnetization prepared-rapid gradient echo (i.e., MPRAGE) using Geodesic information flows (GIF)^11^ to define 142 anatomical brain regions of interest (ROIs). The intracranial mask, cortex, and gray matter (GM) models were extracted from the brain parcellation. A skull surface was generated from a pseudo-computed tomography image.^12^

Sulcal models were extracted from the brain parcellation using a threshold for GM. GM near the surface of the brain was removed by morphologically eroding the intracranial mask and then multiplying this with the GM image.

Vessel models were extracted from the corresponding images (MR + Gad, MRV, MRA, and DSA) as follows. First, a multiscale Sato filter^13^ was applied to enhance vessels on the image. Extracranial blood vessels were removed from consideration by the application of an intracranial mask. The intracranial mask was obtained from the GIF parcellation after coregistration to the vessel scan through an affine transformation. Interactive marching cubes were used to extract the vessel surfaces based on a user-defined intensity threshold. The surface was postprocessed to remove any small unconnected components that were deemed to most likely be noise.

All images and models were coregistered to the MR + Gad image. An affine transformation (rotation, translation, and independent scale) was computed to maximize normalized mutual information between the reference MR + Gad image and the corresponding floating image.^14^ For MRV and MRA, the magnitude scan was used as the floating image and for DSA the bone scan was used as the floating image. Models were coregistered by applying the computed transformation for the corresponding floating image from which the model was generated (i.e., cortex was transformed using the MPRAGE transformation).

Vessel diameter of the DSA meshes was measured from the DSA meshes fit with a centerline using a flux-driven automatic centerline extraction algorithm.^15^ The radius of the DSA meshes was computed as the distance to the centerline for every vertex on each DSA mesh. Meshes are then created by removing vertices corresponding to diameters below 1.0, 2.0, 3.0, and 4.0 mm.

### Computer-Assisted Planning

CAP was performed using EpiNav (University College London, London, United Kingdom) employing a previously described “anatomically-driven multiple trajectory planning” algorithm, that has been shown to generate feasible trajectories faster than expert manual planning. We refer readers to our previous work for an in-depth description of the algorithm.^4^, ^16^ In brief, the user defines a set of target ROIs by selecting anatomic labels from the GIF parcellation based on the multispecialty assessment of the structural and functional neuroimaging, video telemetry-electroencephalogram, semiology, and neuropsychological/psychiatric assessment. If required, an entry ROI also was specified. Candidate target points that were contained within the target ROI and a user-defined distance (≥3 mm) from critical structures were selected.

Potential trajectories (>100,000) were defined by considering all combinations of candidate target points and entry points on the skull segmentation. Potential trajectories were removed if they did not meet minimum drilling angle or length criteria, or did not traverse a specified entry ROI. Next trajectories were removed from consideration if they traversed a critical structure. Trajectories that met these constraints were then optimized on a per-plan basis to ensure all electrode trajectories were 1) ≥10 mm apart from other trajectories; 2) have a low RS, calculated as the cumulative distance from critical structures along the trajectory (see equation); and 3) have a high number of electrode contacts within the GM. The resulting electrode combination for each set of critical structures was then evaluated by the neurosurgeon before implantation. The electrode trajectories were directly exported to the StealthStation S7 NeuroNavigation system (Medtronic Inc., Minneapolis, Minnesota, USA) for stereotactic implantation.

CAP was performed for the following configuration of models considered as critical structures: 1) DSA, 2) MR + Gad, and 3) MRV + A (MRA + MRV) alone or in combination with cerebrospinal fluid- and GM-derived sulcal models. Trajectory recalculations for the same implantation strategies based on the different vascular segmentations took ∼60 seconds. The creation of all other models is automated once a whole brain parcellation is supplied, thereby limiting the amount of technical expertise required. Minimum distance (MD) and overall RS from critical structures were measured for each electrode. In this case, Ad-Tech electrodes (Ad-Tech Medical Instrument Corp., Oak Creek, Wisconsin, USA) were modeled, but the approach is generalizable to all electrode manufacturers as long as the electrode specifications are known. The distance measurements are then able to account for the electrode diameter when generating the RS. RS was calculated using the following equation:

$$RS=\;\{\begin{array}{c} \sum_{i}^{N}\frac{10-Dist\left(i\right)}{N\left(10-3\right)},\;Dist\left(i\right)>3\\ 1+\sum_{i}^{N}\frac{3-Dist\left(i\right)}{3N},\;Dist\left(i\right)\leq 3 \end{array}$$

where *N* is the total number of nodes along the trajectory (*N* = 128) and ί is the index of the individual node.

Vessel diameters from 1 to 4 mm were iteratively removed from the DSA segmentation and RS and MD values for the CAP generated electrodes were recalculated.

### Statistical Analysis

A normality test (Shapiro–Wilk test implemented in R [R Foundation for Statistical Computing, Vienna, Austria]) was performed for each set of metrics to determine whether the data were well modeled by a normal distribution. Each metric that was normally distributed was tested for statistical signification between groups using a paired Student *t* test. For non-normally distributed metrics, a Wilcoxon signed rank test was used to assess statistical significance. In all cases, a Bonferroni correction was applied to account for multiple comparisons (*n* = 3), and *P* < 0.016 was taken as significant.

## Results

All 10 patients (95 electrodes) were included in the quantitative analysis. The distribution of all metrics was determined to be nonparametric. For all further analysis, the use of nonparametric descriptive statistics (median, interquartile range) and nonparametric statistic test (Wilcoxon signed rank test) were used.

**Figure 1:**
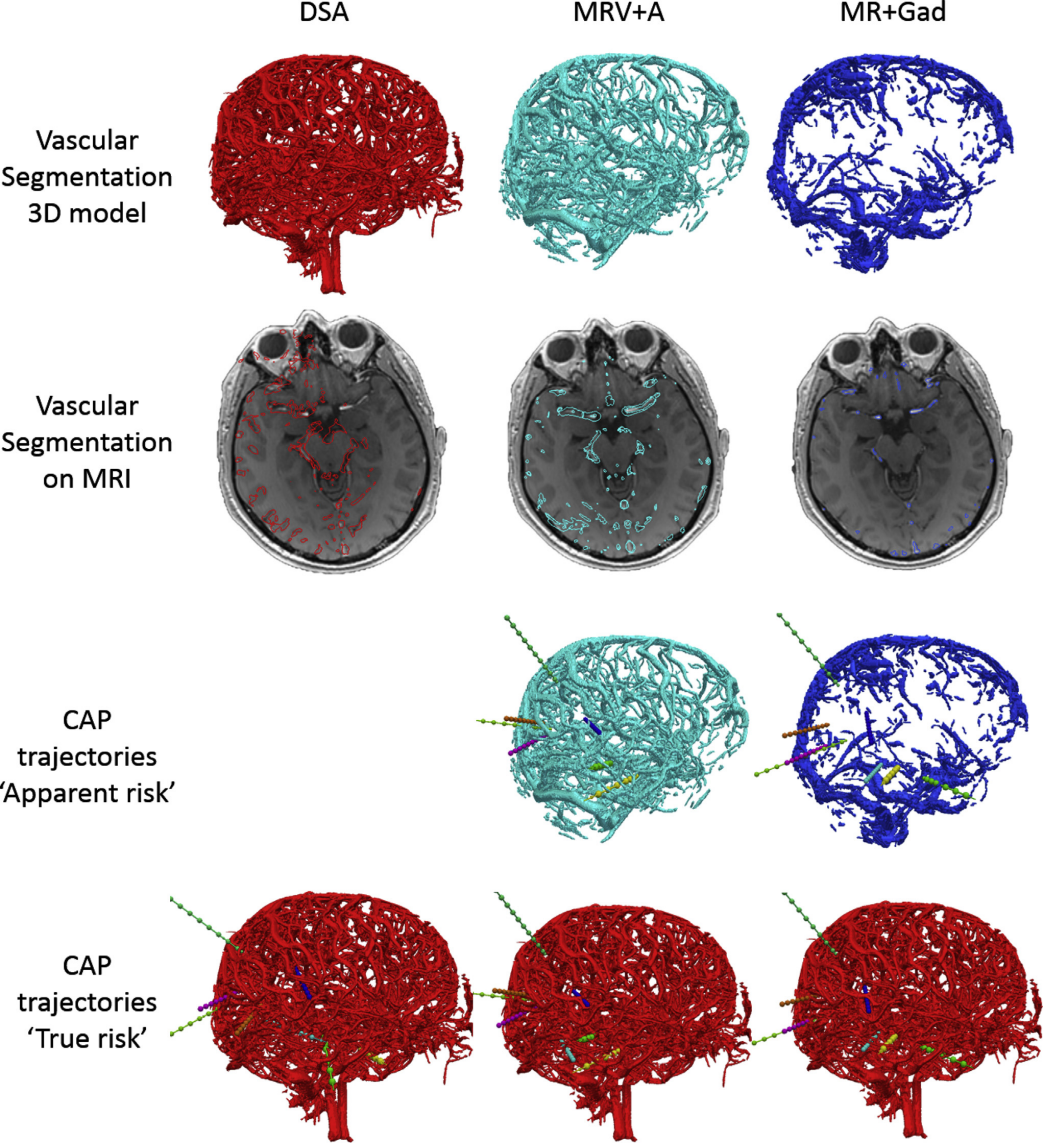
Grid of images providing a representative example of the vessel segmentations derived from the digital subtraction catheter angiography (DSA), magnetic resonance angiography + magnetic resonance venography (MRV + A), and gadolinium-enhanced magnetic resonance imaging (MR + Gad) modalities shown as both 3-dimensional models and an axial slice at the level of the origin of the middle cerebral artery. Computer-assisted planning trajectories planned using the MRV + A and MR + Gad provide the “apparent” risk, which were recalculated using the DSA model segmentation as the gold standard and therefore representing the “true” risk.

**Figure 2:**
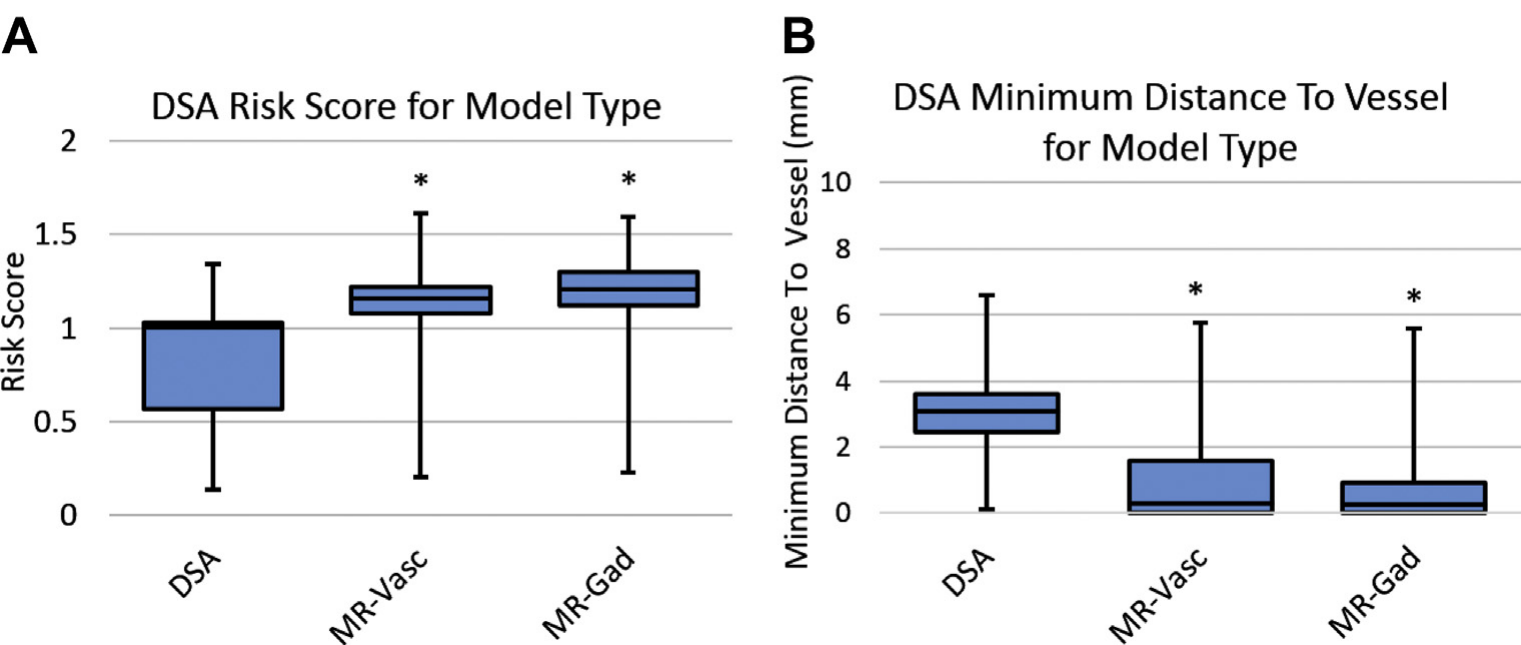
(**A**) “True” risk score and (**B**) Minimum distance to blood vessel using digital subtraction catheter angiography (DSA) to determine blood vessel location for each model combination considered during computer-assisted planning (CAP). *Indicates values that were statistically significantly different compared to using DSA with no sulci applied for CAP. MR-Vasc, magnetic resonance angiography + magnetic resonance venography; MR-Gad, gadolinium-enhanced magnetic resonance imaging.

**Figure 3:**
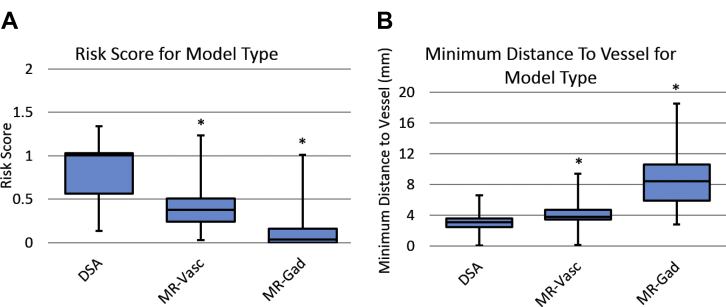
(**A**) “Apparent” risk score and (**B**) minimum distance to blood vessel using the specified model to determine blood vessel location for each model combination considered during computer-assisted planning (CAP). *Indicates values that were statistically significantly different compared with using digital subtraction catheter angiography (DSA) with no sulci for CAP. MR-Vasc, magnetic resonance angiography + magnetic resonance venography; MR-Gad, gadolinium-enhanced magnetic resonance imaging.

A representative example of the vessel segmentations and effects on “apparent” and “true” risk for CAP generated trajectories are shown in Figure 1. The metrics for each model type when recomputed using the blood vessels obtained from DSA (considered as the gold standard) is shown in Figure 2. The metrics computed for the models used to guide CAP (note for the DSA models these values are equivalent) is shown in Figure 3.

### Utility of Sulcal Models

We tested the statistical significance of using sulcal models against not using sulcal models for each blood vessel imaging modality studied. The use of sulcal models did not significantly change the RS. For further analysis, we consider only trajectories generated with no sulci.

### Vessel Model Suitability

As shown in Figure 2, using DSA to compute the CAP plan resulted in the lowest “true” RS and greatest MD. Figure 3 displays the metrics computed using the same models as used for CAP, which corresponds to the “apparent” RS, i.e., values provided to the user when planning with CAP. For both MR + GAD and MRV + A, these values are statistically significantly lower for RS and MD. This indicates MR-based vascular segmentations result in a falsely lower “apparent” RS during CAP than the assumed “true” RS provided by the DSA for the same electrode trajectories.

Furthermore, we computed the Pearson correlation between the “apparent” RS using MRV + A and T1 + GAD with the “true” RS from DSA and found values of R^2^ = 0.003 and R^2^ = 0.001, respectively. This poor correlation demonstrates that it is not possible to infer the presence of vasculature and therefore “true” RS, when performing CAP with MRV + A or MR + Gad alone.

### Vessel Model Equivalence

**Figure 4:**
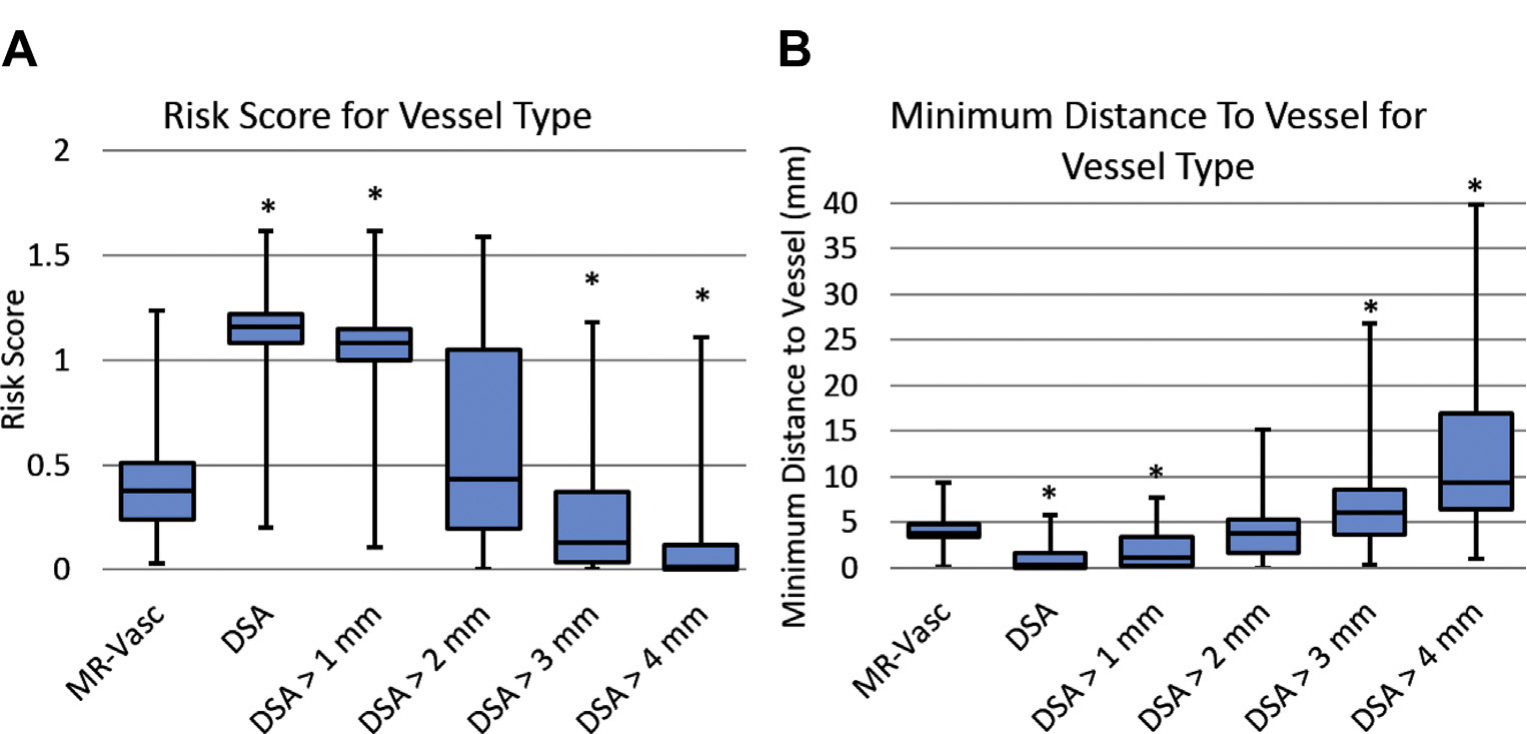
(**A**) Risk score and (**B**) minimum distance to blood vessel using the specified vessel type for electrodes computed by computer planning with magnetic resonance angiography + magnetic resonance venography (MRV + A) as the critical structures. *Indicates values that were statistically significantly different compared with using MRV + A. MR-Vasc, magnetic resonance angiography + magnetic resonance venography; DSA, digital subtraction catheter angiography.

**Figure 5:**
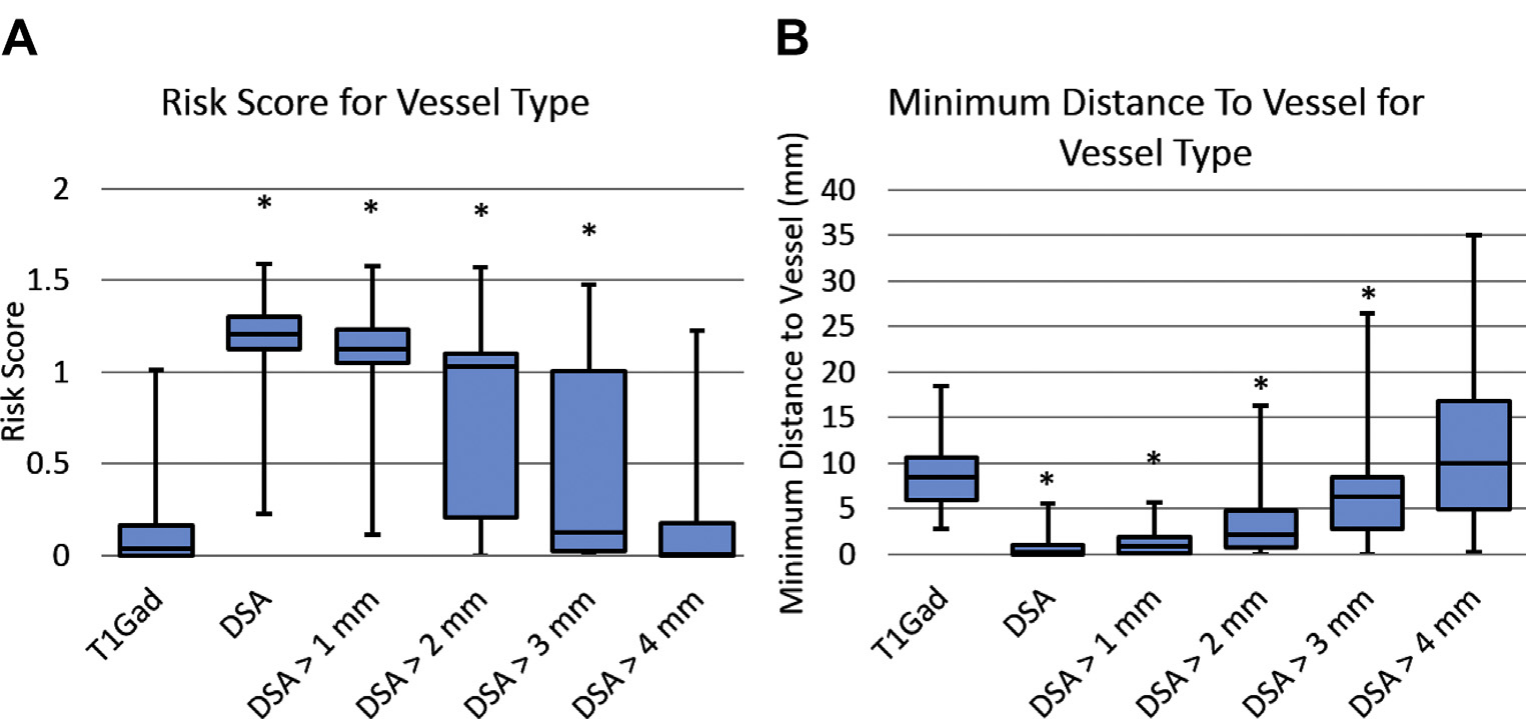
(**A**) Risk score and (**B**) minimum distance to blood vessel using the specified vessel type for electrodes computed by computer planning with gadolinium-enhanced magnetic resonance imaging (MR + Gad) blood vessels as the critical structures. *Indicates values that were statistically significantly different compared to using MR + Gad. T1Gad, gadolinium-enhanced magnetic resonance imaging; DSA, digital subtraction catheter angiography.

Electrodes planned with MR + Gad or MRV + A were assessed using the “true” RS and MD to DSA vessels at different diameters (computed using a centerline algorithm as described in the section “Model Generation”). For MRV + A, the RS and MD were statistically equivalent to the DSA segmentation when vessels ≤2 mm were removed and returned significantly better metrics than using a DSA when vessels ≤3 mm were removed (see Figure 4). For MR + Gad, the RS and MD were statistically equivalent to DSA when vessels <4 mm were removed from consideration (see Figure 5).

## Discussion

SEEG is increasingly being used in the presurgical investigation of patients with drug-resistant focal epilepsy when noninvasive investigations are inconclusive or discordant.^17^ Implantation schema outlining the number of electrodes as well as the ROIs to be sampled is based on a multidisciplinary assessment of the clinical history, ictal semiology, video-telemetry, imaging, and neuropsychological and psychiatric assessments. CAP algorithms have been used as clinical decision support software to optimize the precise planning of SEEG trajectories and have been shown to improve trajectory planning metrics.^4^, ^16^, ^18^, ^19^ Meta-analysis of the complications associated with SEEG implantation has shown the risk of morbidity per electrode to be 1 in 287 electrodes, which equates to 1 in 29 patients.^2^ It is vital that this risk is minimized. Previous studies have shown that there is wide variation in SEEG planning, vascular segmentation,^10^ and stereotactic implantation methods.^20^ Safe SEEG is dependent on optimal trajectory planning through avascular corridors and accurate stereotactic implantation. Different methods for stereotactic implantation include frame-based,^21^, ^22^ frameless,^23^, ^24^ custom-jig,^25^ and robot-assisted^26^, ^27^, ^28^ techniques. Pooled entry and target point accuracies from a random-effects meta-analysis have been calculated to be 1.6 mm and 2.3 mm, respectively.^20^

In a large study reporting 500 SEEG implantations, Cardinale et al.^21^ implemented a minimum safety margin of 3 mm from intracranial vasculature during trajectory planning based on their previous implantation accuracies. The ability to implement such a margin, however, is dependent on the method of vascular imaging employed and the corresponding vasculature segmentation. To date, there have been no reports identifying the minimum vessel diameter that, if transgressed by an electrode, would lead to intracerebral hemorrhage. A single study, using susceptibility-weighted imaging acquisitions in 13 patients undergoing deep brain stimulation, SEEG, and laser interstitial thermal therapy reported 63 vessel-electrode conflicts, of which only 13 were identifiable on MR + Gad.^29^ The mean size of vessel conflict from susceptibility-weighted imaging was 1.49 ± 0.46 mm (mean ± standard deviation) compared with 2.01 ± 0.52 mm (mean ± standard deviation) from MR + Gad. In this series, no patients were reported to have an intracerebral hemorrhage and, as such, the upper limit for vessel size conflict resulting in hemorrhage could not be determined.

DSA is the gold standard for imaging of intracranial vasculature and provides detailed visualization of vessels <1 mm in diameter. It is, however, also an invasive procedure requiring radiation exposure and carries risks of stroke and puncture-site morbidity.^30^ Furthermore, the detailed imaging provided by DSA may be overly restrictive, preventing feasible SEEG trajectories once safety margins are applied. A balance is therefore required between adequately visualizing vessels for safe SEEG and being able to identify sufficient avascular corridors through which trajectories can be planned.

CAP algorithms increasingly have been used to improve the potential safety and efficiency of SEEG planning^3^, ^18^ with external expert feasibility ratings achieving those of manually planned electrodes.^4^ CAP algorithms are able to optimize trajectories based on user-defined constraints. These include entry and target ROIs, intracerebral trajectory length, drilling angle, GM sampling ratio, distance from other planned electrodes, MD, and RS. CAP algorithms calculate MD and RS metrics from the segmented vascular models used during planning.^16^ CAP works to avoid critical structures represented as triangular mesh elements; this allows for rapid collision detection between trajectories (lines) and the critical structures (triangles).^31^ However, this limits CAP to only avoid structures that can be identified and segmented from preoperative imaging. Previous work has shown the majority of infeasible CAP trajectories are near to blood vessels that were not accurately segmented.^3^, ^4^ The vesselness filter used in this study^13^ is only one method of several that have been presented in the literature to extract blood vessels.^32^, ^33^, ^34^ Each vessel segmentation method will have its own limitations to accurately segment blood vessels, which in turn affect the ability of CAP to compute safe electrode trajectories. In general, vascular imaging capable of resolving smaller vessels with higher contrast signal, such as DSA, will have a better segmentation accuracy compared with imaging that cannot resolve small vessels (such as MR + Gad) across segmentation algorithms. Manual planning is less affected by this phenomenon, however, as a large proportion of the unsegmented vasculature will still be visible during the review of the raw imaging acquisition.

CAP-generated trajectories from MR + Gad and MRV+A resulted in statistically greater “true” RS and closer MDs to vasculature. The implication is therefore that CAP requires DSA vessel segmentations to return trajectories with the lowest RS and greatest MD. If this has a direct clinical benefit, it will be difficult to determine due to the low incidence of hemorrhage, the prohibitively large sample size required for the comparison, and the significant variability in trajectory planning. Furthermore, as the minimum vessel diameter for consideration during SEEG has yet to be identified, we have also iteratively removed vasculature of varying diameters to determine the level at which MRV + A and MR + Gad RS and MD become equivalent to DSA. Here, we find that if vessels <2 mm and <4 mm are considered to be inconsequential then MRV + A and MR + Gad, respectively, are equivalent to DSA.

**Figure 6:**
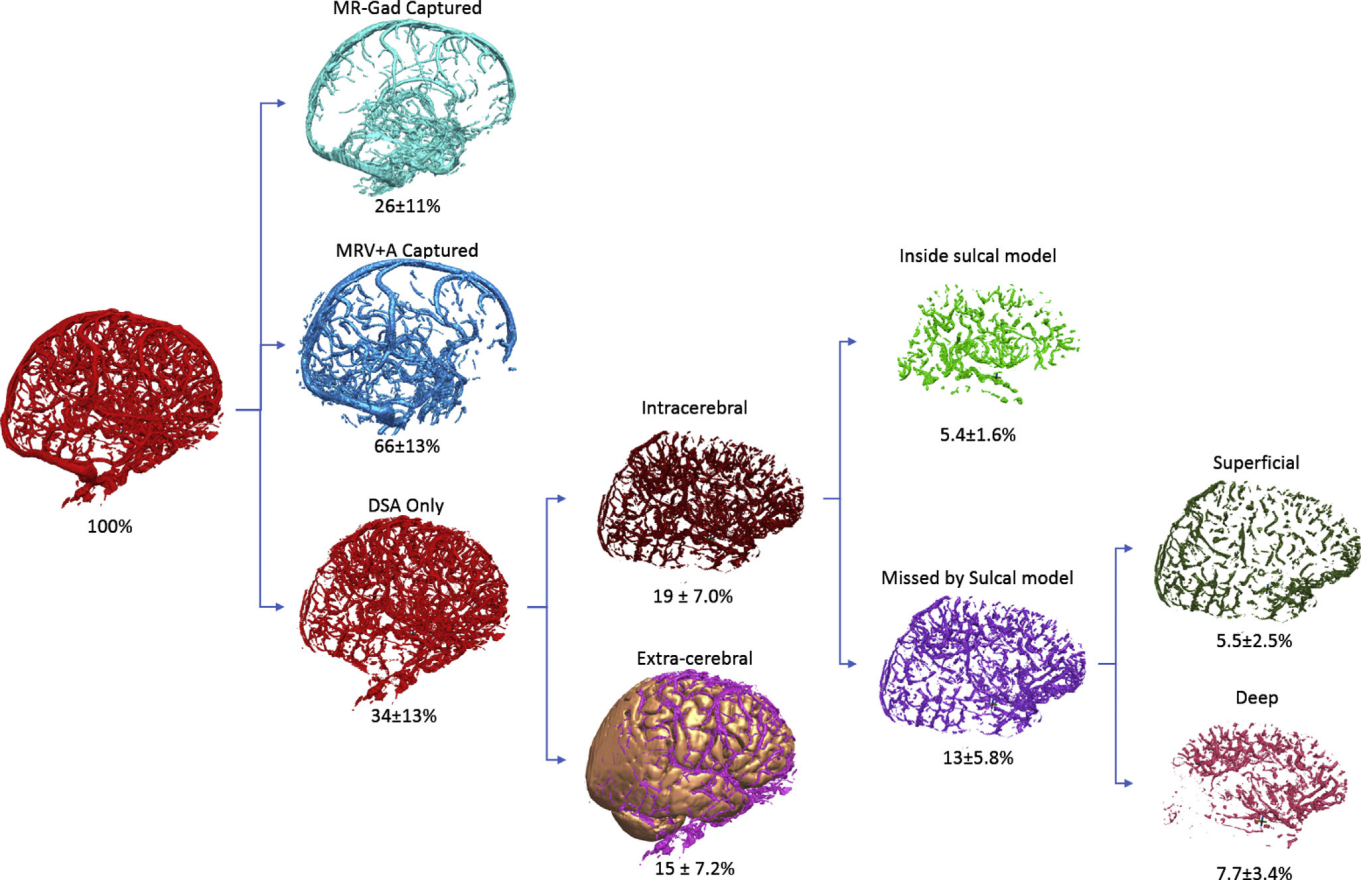
Proportion of blood vessels by anatomical region. Of all the blood vessels segmented from the digital subtraction catheter angiography (DSA), 2/3 are also present on magnetic resonance angiography + magnetic resonance venography (MRV + A). Those present only on DSA were further divided into those within the brain (intracerebral) or on the surface of the brain (cortical surface). Of the intracerebral vessels that were captured by the DSA, but not by the MRV + A, 5.4% were captured by the sulcal models. The remaining vessels that were missed by the sulcal model were either superficial, i.e., overlying the top of the sulcal or deep, representing vessels on the medial side of the target structures. MR-Gad, gadolinium-enhanced magnetic resonance imaging.

Although cadaveric dissections reveal small blood vessels within deep sulci, we did not find evidence that preventing sulcal transgression significantly affected RS and MD from vasculature. This is due to DSA being able to resolve vessels within sulci and hence, when used in combination with CAP, identify avascular corridors through sulci that maintain the predefined safety margin of 3 mm. When evaluating the distribution of segmented DSA vessels according to brain anatomy (Figure 6), we found an equal chance of blood vessels being within sulci as within deep cortical tissue, i.e., at the target sites, which may explain why the inclusion of a sulcal model did not significantly change CAP RS. Interestingly, a previous study from the deep brain stimulation literature that did not use vascular segmentation found a 10-fold increase in hemorrhage rates during deep brain stimulation procedures when crossing sulci.^35^ We conclude therefore that when DSA is used for planning, crossing sulci does not increase the RS.

### Limitations

We made 2 implicit assumptions in this study that may limit the validity of our findings. First, we assumed that DSA is a gold standard blood vessel segmentation model. We assumed DSA accurately captures all significant blood vessels present in the brain and that measures of blood vessel diameter performed on DSA are reflective of the true size of the blood vessel. Although DSA is the most accurate blood vessel image acquisition we evaluated, small blood vessels may still be missed during segmentation. In addition, due to partial volume effects on DSA (with voxel resolution of approximately 0.5 mm^3^) measuring blood vessel diameter below 1 mm is not accurate with the current segmentation algorithm.

Second, implementing an RS assumes that the closer an electrode is to a blood vessel, the greater the hemorrhage risk. Although intuitively one would expect this to be the case and reflects current neurosurgical practice, this has not been validated. Similarly, we also do not assign a relative risk or likelihood of hemorrhage based on the size or position of blood vessels, as there is no evidence in the literature to quantitatively model this. Small blood vessels may have a reduced risk of causing hemorrhages or the volume of hemorrhage from small vessels may be masked by the electrode artifact and/or be too small to be detected on postoperative computed tomography. Based on the DSA imaging that was acquired, we are also unable to distinguish whether small vessels are arterial or venous, and composition of the walls of the vessels is also likely to impact on whether the vessel is deflected or transgressed during electrode conflict. Due to the paucity of evidence in the literature, we are unable to infer hemorrhage risk associated with vessel size and type. Other factors that are likely to contribute to hemorrhage risk include the biomechanical properties of the electrode and the use of an introducing stylet, although this is not the focus of this study.

Finally, we accept that the conclusions of the study are derived from the image acquisition protocols employed at our institution. We make our imaging protocols and CAP software (EpiNav) available for use, free of charge, to collaborating centers to help standardize SEEG trajectory planning in an objective and systematic fashion.

## Conclusions

There is an increasing shift toward SEEG for the intracranial investigation of drug-resistant focal epilepsy. There is significant variation in the planning, vascular segmentation, and stereotactic implantation methodology between epilepsy surgery centers. Accurate visualization of intracranial vasculature is key to safe trajectory planning. CAP has been shown to improve trajectory safety metrics, but this is dependent on the vascular segmentation used during planning. MR-based vascular segmentations during CAP planning result in greater RS. MRV + A and MR + Gad RS and MD metrics become equivalent to DSA if vessels ≤2 mm and ≤4 mm, respectively, are not considered planning. To our knowledge, this is the first study directly comparing different vascular modalities in the same patients and quantifying the impact on CAP. Future studies identifying the critical size at which vascular conflicts result in hemorrhage will be particularly helpful in guiding future clinical practice.

## Declaration of Competing Interest

Conflict of interest statement: We acknowledge funding support by National Institute for Health Research University College London Hospitals NHS Foundation Trust/University College London (NIHR UCLH/UCL) Biomedical Research Centre and senior investigator schemes and Wellcome Trust (WT106882)/Wellcome/EPSRC [203145Z/16/Z].

## Supplementary Material

### Image Acquisition

Magnetic resonance imaging (MRI) data were acquired on an MR750 3T, with maximum gradient strength of 50 mT/m and maximum slew rate of 200 T/m/s, with a 32-channel head coil. A coronal oblique 3-dimensional (3D) T1-weighted magnetization-prepared rapid gradient echo image was acquired with a 1-mm^3^ isotropic resolution and the following acquisition parameters: repetition time (TR)/echo time (TE)/inversion time = 7.4/3.1/400 milliseconds; flip angle 11°; field of view (FOV) 224 × 256 × 256 mm (anterior-posterior × right-left × inferior-superior); acquisition matrix 224 × 256 × 256; parallel imaging acceleration factor of 2.

Vascular imaging included a postgadolinium T1-weighted (MR + Gad) and phase-contrast magnetic resonance angiography (MRA) and magnetic resonance venography (MRV) scans. The straight axial postgadolinium T1-weighted scan was acquired with a 3D fast spoiled gradient echo sequence with a FOV of 256 × 256 × 184 mm and an acquisition and reconstruction matrix of 256 × 256 × 184 (TE/TR 3.0/7.3 milliseconds; flip angle 11°). Both the MRA and MRV were performed using a 3D phase-contrast sequence with a FOV of 220 × 220 × 148.8 mm and an acquisition matrix of 384 × 256 × 124, for a reconstructed voxel size of 0.43 × 0.43 × 0.60 mm (flip angle 8°; parallel imaging acceleration factor 2). To highlight the arteries, MRA was performed with a velocity encoding of 80 cm/second (TE/TR 4.0/9.3 milliseconds). For sensitivity to the venous circulation, the MRV was performed with a velocity encoding of 15 cm/second (TE/TR 4.8/26.4 milliseconds), fat suppression, and a saturation band inferior to the FOV to null inflowing blood and prevent aliasing.

DSA imaging was performed through femoral puncture under local anesthesia. Using fluoroscopic guidance, an endovascular catheter was inserted into the ipsilateral internal carotid artery and/or vertebral artery. A 3D acquisition was performed before and after intra-arterial injection of contrast agent. Digital subtraction was then performed between the pre- and postcontrast images to provide a bone-stripped angiogram. Images were then reconstructed with voxel sizes of 0.5 × 0.5 × 0.5 mm.
